# Mouse Estrous Cycle Identification Tool and Images

**DOI:** 10.1371/journal.pone.0035538

**Published:** 2012-04-13

**Authors:** Shannon L. Byers, Michael V. Wiles, Sadie L. Dunn, Robert A. Taft

**Affiliations:** 1 Reproductive Sciences R&D, The Jackson Laboratory, Bar Harbor, Maine, United States of America; 2 Technology Evaluation and Development, The Jackson Laboratory, Bar Harbor, Maine, United States of America; National Cancer Institute, United States of America

## Abstract

The efficiency of producing timed pregnant or pseudopregnant mice can be increased by identifying those in proestrus or estrus. Visual observation of the vagina is the quickest method, requires no special equipment, and is best used when only proestrus or estrus stages need to be identified. Strain to strain differences, especially in coat color can make it difficult to determine the stage of the estrous cycle accurately by visual observation. Presented here are a series of images of the vaginal opening at each stage of the estrous cycle for 3 mouse strains of different coat colors: black (C57BL/6J), agouti (CByB6F1/J) and albino (BALB/cByJ). When all 4 stages (proestrus, estrus, metestrus, and diestrus) need to be identified, vaginal cytology is regarded as the most accurate method. An identification tool is presented to aid the user in determining the stage of estrous when using vaginal cytology. These images and descriptions are an excellent resource for learning how to determine the stage of the estrous cycle by visual observation or vaginal cytology.

## Introduction

Identifying the stage of estrous is useful for choosing mice that will mate when paired with a male (to produce timed pregnancy or pseudopregnancy) or tracking the stage of estrous as a variable that may affect research. In the mouse, the estrous cycle is divided into 4 stages (proestrus, estrus, metestrus, and diestrus) and repeats every 4 to 5 days unless interrupted by pregnancy, pseudopregnancy, or anestrus. The cycle has been described in detail elsewhere and the reader is directed to these references for a full description [1], [2].

Changes occurring in the mouse estrous cycle are evident in the animal's physiology and anatomy. These changes can be detected using a variety of methods to determine the stage of estrous and include evaluating vaginal cytology [1], [3], [4], [5], measuring electrical impedance [6], biochemical analysis of urine [7], and visual observation of the external genitalia [8]. Evaluating vaginal cells is accepted as the most accurate method for identifying all stages of the estrous cycle however, it is relatively labor intensive requiring vaginal cells to be collected, transferred to a glass slide, air dried, stained, and viewed. The vaginal cytology method is best used when all 4 stages of the estrous cycle need to be identified. Alternatively, the electrical impedance of the vaginal epithelial cell layer during proestrus is higher than during other stages and can be measured using a probe to identify mice that are in proestrus. One limitation, however, is that other estrous stages cannot be distinguished using this method. The method for determining the stage of estrous by visual observation described by Champlin, et al [8] is considerably quicker than the cytology or electrical impedance methods. Once a person is trained, approximately 100 females can be evaluated in 10–15 minutes, no extra equipment is required and evaluation can be completed without leaving the vivarium. Additionally, with visual evaluation, there is no risk of inducing pseudopregnancy or damaging the vaginal epithelium as there may be with inserting a swab for collecting cells or a probe for measuring electrical conductance. Mice identified as being in proestrus or estrus are likely to be receptive to males making this method well suited for identifying mice likely to mate, facilitating timed mating. In the Jackson Laboratory's Reproductive Sciences group greater than 90% of mice “selected” as being in proestrus or estrus successfully mate overnight.

Pseudopregnant outbred albino (CD-1) or hybrid agouti mice are often used as embryo recipients and detecting proestrus/estrus in these strains is straightforward. However, coat color is not a primary concern when choosing embryo recipient strains or strains for timed matings or artificial insemination. In these situations it is useful to be able to identify stage of estrus in strains of different coat colors.

Learning the visual identification and cytology methods requires practice. Presented here are tools to help the user identify stages of the estrous cycle in albino, black, and agouti strains when using the visual observation method. The visual observation method is best suited for identifying animals that are in proestrus or estrus for the purpose of producing timed matings or pseudopregnant mice. For those needing to accurately identify all stages of the estrous cycle, the vaginal cytology method is recommended and an estrous cycle identification tool is provided to aid users in determining the correct stage based on the type and proportion of cells in the smear.

## Materials and Methods

### Ethics Statement

The Institutional Animal Care and Use Committee of The Jackson Laboratory approved all procedures used in this study and all mice were maintained at The Jackson Laboratory (Bar Harbor, ME, USA) in accordance with all institutional protocols and the Guide for the Care and Use of Laboratory Animals.

### Mice

Mice from the following strains were used: C57BL/6J (stock no. 000664), CByB6F1/J (stock no. 100009), and BALB/cByJ (stock no. 001026). All mice were housed in a non-barrier facility with a photoperiod of 14 hours of light and 10 hours of dark (lights on at 5:00AM). Mice were housed in polycarbonate duplex mouse cages, 5 per side with pine shaving bedding, and were provided food (LabDiet® 5K52, St. Louis, MO, USA) and water (acidified) ad libitum. Adult mice between the ages of 9–13 wks (C57BL/6J), 14–23 wks (CByB6F1/J), and 8–12 wks (BALB/cByJ) were used.

### Determining stage of estrous cycle

Representative photographs and micrographs for each stage of the estrous cycle were obtained following these steps: 1. a preliminary observation was made about the stage of the estrous cycle by assessing the vaginal opening of each mouse, 2. the stage of the estrous cycle was verified by vaginal cytology, 3. stage of estrous cycle was confirmed by mating mice overnight and checking for ovulation the following morning as described later.

These steps can also be used to learn the visual method and train the eye to identify each stage. Proestrus and estrus are easier to identify by visual observation than metestrus and diestrus. Coat color and skin pigmentation can make it more challenging to evaluate some strains. It is easier to observe changes in agouti and albino strains than in black strains where changes to the vaginal opening are more subtle.

### Visual Method

To evaluate the stage of the estrous cycle by visual observation, each mouse was held by the tail with the forepaws resting on a cage lid. The vaginal opening of each female was evaluated based on the criteria described by Champlin, et al. A digital image of each mouse was taken using a DSCF707 Cyber-shot digital camera (Sony, Japan). Additional lighting was supplied for photographs by fiber optic lights (Fiber Lite MI-150, Dolan-Jenner Industries, Boxborough, MA).

When evaluating stage of the estrous cycle using the visual method, it is important to always evaluate animals in the same area with respect to room lighting. The table or workstation should always face the same direction and there should be sufficient light available. The light source is also important to consider because it can change the perceived color of vaginal tissues and make evaluation difficult. Portable lights can be purchased and attached to workstations and moved as needed. However, LED lights should be avoided because they have a purple hue that makes visual detection challenging. Battery operated 4W fluorescent lamps (Maverick, Edison, NJ) were used in the vivarium for this study. In the laboratory, 32W Sylvania Octron fluorescent ceiling lights (Sylvania, Danvers, MA) were used for lighting.

The vaginal opening of mice in proestrus is characterized by swollen, moist, pink tissue. The opening is wide and there are often wrinkles or striations along the dorsal and ventral edges. As the mouse enters estrus the vaginal opening becomes less pink, less moist, and less swollen. Metestrus is characterized by a vaginal opening that is not open wide, not swollen, and white cellular debris may be visible. In diestrus, the vaginal opening is small and closed with no tissue swelling.

### Vaginal Cytology Method

A vaginal swab was collected using a cotton tipped swab (Puritan Medical Products Company, LLC Guilford, ME) wetted with ambient temperature physiological saline and inserted into the vagina of the restrained mouse. The swab was gently turned and rolled against the vaginal wall and then removed. Cells were transferred to a dry glass slide by rolling the swab across the slide. The slide was air dried and then stained with approximately 400 µL of stain (Accustain, Sigma-Aldrich, St. Louis, MO) for 45 seconds. The slides were rinsed with water, overlaid with a coverslip, and viewed immediately at 200× magnification under bright field illumination. The stage of the estrous cycle was determined based on the presence or absence of leukocytes, cornified epithelial, and nucleated epithelial cells according to Felicio, et al [9].

When the female is in proestrus, mostly nucleated and some cornified epithelial cells are present. Some leukocytes may be present if the female is in early proestrus. As the stage of the cycle advances to estrus, mostly cornified epithelial cells are present. If the cycle is not interrupted by pregnancy, pseudopregnancy, or other phenomena, metestrus will begin. Metestrus is a brief stage when the corpora lutea form but fail to fully luteinize due to a lack of progesterone. The uterine lining will begin to slough and evidence of this is seen in the form of cornified eipithelial cells and polymorphonuclear leukocytes present in vaginal swabs. Some nucleated epithelia cells will also be present in late metestrus. Diestrus is the longest of the stages lasting more than 2 days. Vaginal swabs during diestrus show primarily polymorphonuclear leukocytes and a few epithelial cells during late diestrus. Leukocytes remain the predominant cell type having removed cellular debris. The cycle then repeats.

### Determining if ovulation occurred

After the stage of the estrous cycle was determined, each mouse was paired with one CByB6F1/J male and the following morning the presence or absence of a vaginal plug was noted and each mouse was euthanized by cervical dislocation. The contents of the oviducts were flushed using M2 media into a petri dish to look for oocytes as evidence that ovulation had occurred.

### How to Use the Estrous Cycle Identification Tool

**Figure 1:**
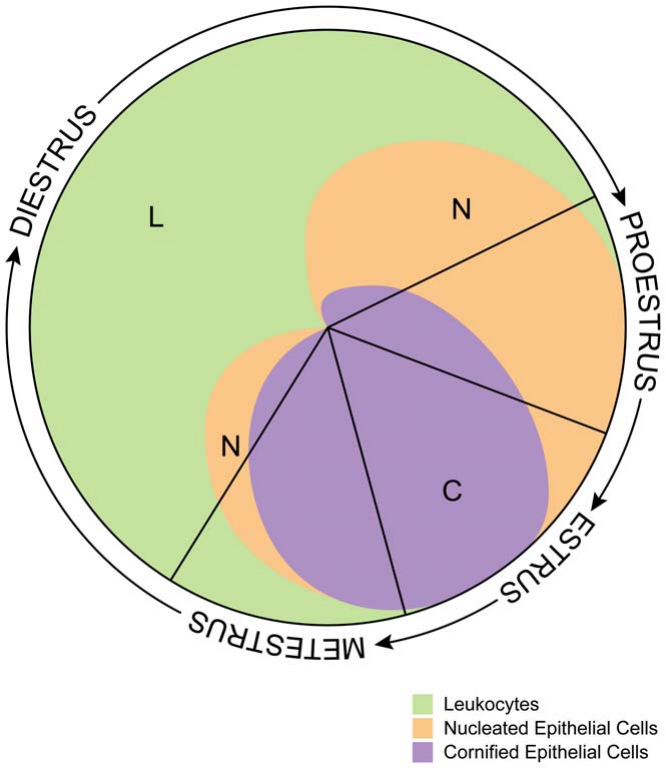
Estrous cycle stage identification tool. This tool is a visual representation of cell types and relative proportion of each type present during the four stages of the estrous cycle. The lines in the circle mark where the stage of estrous changes. The size of each quadrant (between 2 lines) is a rough estimate of the length of each stage. The total cycle takes about 4–5 days. To use this chart, vaginal cells are examined and the relative number of each cell type is determined. Then, an imaginary arrow is placed on the chart with the end on the center of the chart like a hand on a clock. The arrow is moved clockwise until the cell types and proportion appear under the arrow. Once the arrow is placed, it points to the corresponding stage of estrous. For example, an arrow in the position of 9 o'clock represents a vaginal smear with all leukocytes and a mouse in diestrus. An arrow at 3 o'clock represents a smear with approximately half cornified epithelial and half nucleated epithelial cells and a mouse in proestrus.

The estrous cycle identification tool was developed using qualitative data from the literature [5], [9], [10] for the proportion of each cell type in a smear. A graphical representation of the existing data was created to represent the typical changes in cell types that occur during the entire estrous cycle. The continuous changes in cell types (leukocytes, nucleated epithelial, and cornified epithelial) occurring during the estrous cycle result in the lack of clear demarcations between stages and can make it difficult to determine the stage of the estrous cycle. For example, the vaginal cytology of a mouse in estrus is characterized by many cornified epithelia cells. However, if the mouse is in early estrus, nucleated epithelial cells may also be present. Presented here (Figure 1) is an estrous cycle identification tool that shows the changes in cell populations during the entire cycle. The estrous cycle identification tool makes it clear what cells types are present at each point of the cycle, including the transitional phases between each stage.

The estrous cycle identification tool is a visual aid that shows the 4 estrous stages and the relative proportion of cells present in each stage. Each cell type is shown in a different color. The name of each stage of the estrous cycle is shown on the outside of the circle progressing clockwise from one stage to the next. The 4 quadrants are different sizes to represent a rough estimate of how much time is spent in each stage of the estrous cycle.

To use the estrous cycle identification tool, collect cells using the vaginal cytology method described and view them using a compound microscope. Identify the cell types present on the slide and note the relative proportion of each cell type. For example, there may be all leukocytes on the slide or there may be about half cornified epithelial and about half nucleated epithelial cells. Next, look at the estrous cycle identification tool (Figure 1) and place an imaginary arrow on the chart with the end on the center of the chart like a hand on a clock. The arrow is moved clockwise until the cell types and proportion appear under the arrow. Once the arrow is placed, it points to the corresponding stage of estrous.

This tool makes it easy to determine the stage of the cycle when vaginal cytology is used. The relative amount and type of cells present during early proestrus and late metestrus are similar. The nucleated epithelial cells in proestrus are often well-formed, but are often irregularly shaped and vacuolated in metestrus [3]. Alternatively, early proestrus and late metestrus can be distinguished using the visual method.

## Results

**Figure 2:**
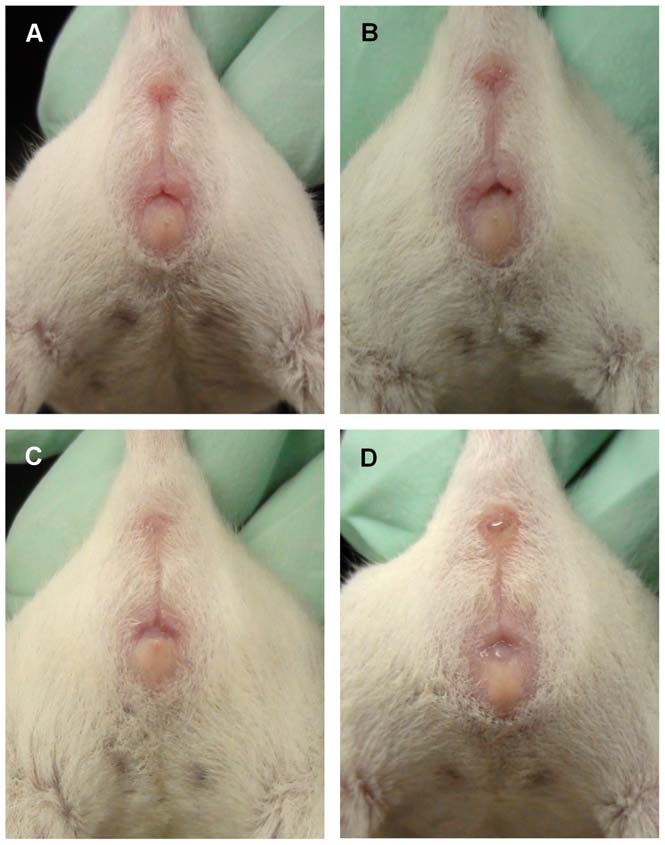
Four stages of estrous in BALB/cByJ mice. The four stages of estrous are shown for an albino strain (proestrus (A), estrus (B), metestrus (C), diestrus (D)).

**Figure 3:**
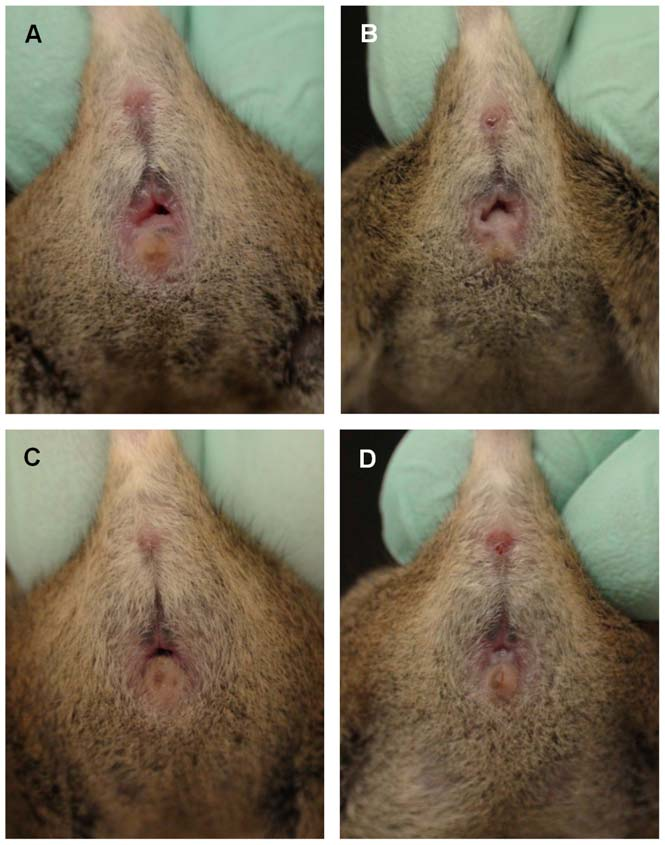
Four stages of estrous in CByB6F1/J mice. The four stages of estrous are shown for an agouti strain (proestrus (A), estrus (B), metestrus (C), diestrus (D)).

**Figure 4:**
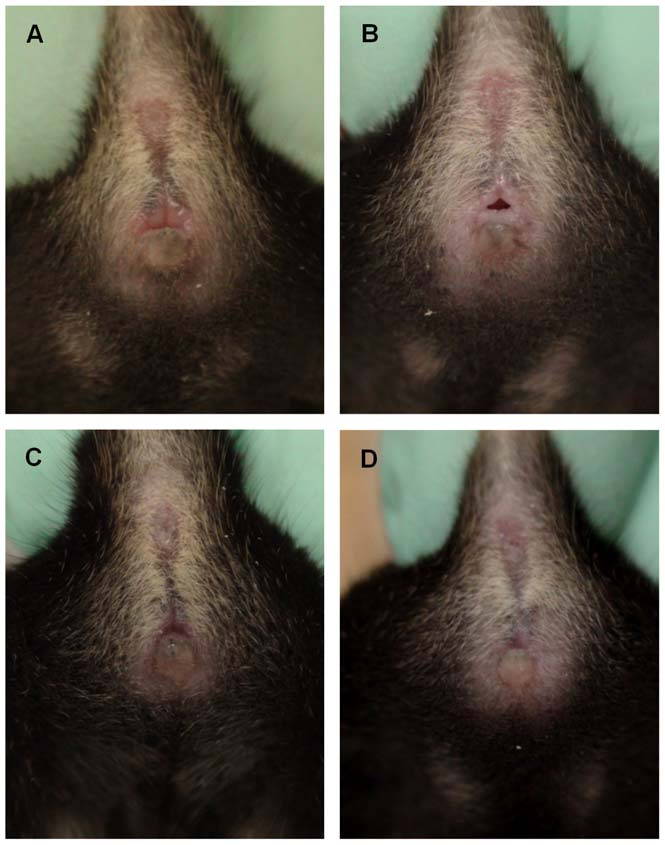
Four stages of estrous in C57BL/6J mice. The four stages of estrous are shown for a non-agouti black strain (proestrus (A), estrus (B), metestrus (C), diestrus (D)).

**Figure 5:**
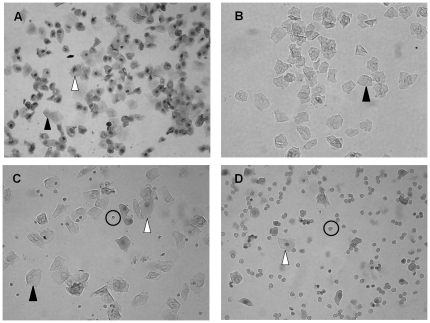
Vaginal cytology representing each stage of estrous. Three cell types are identified: leukocytes (circle), cornified epithelial (black arrow), and nucleated epithelial (white arrow). Stages of estrous include proestrus (A), estrus (B), metestrus (C), diestrus (D).

Representative photographs (Figures 2, 3, and 4) and images (Figure 5) of each estrous stage for 3 strains are presented. All mice were visually evaluated and the estrous cycle stage of each was confirmed by vaginal cytology. We evaluated approximately 50 mice per strain and chose representative images that were clear and accurate in depicting each stage. The images in Figures 2, 3, and 4 are representative of the different stages. For each stage in each strain, the photograph and micrograph are from either one mouse or are compiled from two mice.

For the mice in the photographs shown, all proestrus and estrus mice had oocyte or cumulus oocyte complexes in their oviducts as evidence of ovulation. All metestrus and diestrus mice did not have oocytes in their oviducts.

For the mice in the photographs shown for each strain, the proestrus and estrus mice had vaginal plugs present after being housed overnight with a CByB6F1/J male with the exception of the CByB6F1/J estrus mouse (shown in Figure 3B). A copulatory plug was not found in this female, but oocytes were found in the oviduct indicating ovulation had occurred and corroborating the finding that she was in estrus. The male may have failed to mate or the plug fell out before it could be observed. All metestrus and diestrus mice shown in the photographs did not have vaginal plugs after being housed overnight with the exception of the metestrus CByB6F1/J (shown in Figure 3C) and the metestrus C57BL/6J mouse (shown in Figure 4C). These mice both had plugs. One explanation is these mice were in late estrus/early metestrus at the time of mating and the male mated immediately as they were was introduced. No cumulus oocyte complexes were found in the oviduct of these mice the next morning indicating the metestrus evaluation was correct. Another explanation is overzealous males that mated without regard to the stage of estrous.

## Discussion

Timed matings are useful when embryos of a precise stage are need, when it is necessary to accurately predict when mice will give birth, or to produce pseudopregnant mice for embryo transfer or artificial insemination. It is common practice to randomly mate mice to produce timed matings or pseudopregnancies. However, random mating is inefficient. Using the visual observation method, the number of mice that successfully mate is dramatically increased. Determining the stage of the estrous cycle by visual observation is simple and easy to learn. The visual detection method is best to identify proestrus and estrus females. It can be difficult to distinguish the other stages using this method alone. If all stages must be identified accurately, the vaginal cytology method using the estrous cycle identification tool is recommended.

The methods and images presented are to aid in learning how to determine the stage of the estrous cycle. The images from different coat color strains will be useful for those learning the visual observation method with any strain. The estrous cycle identification tool will aid those using the vaginal cytology method.
